# Cardiac MLC2 kinase is localized to the Z-disc and interacts with α-actinin2

**DOI:** 10.1038/s41598-019-48884-w

**Published:** 2019-08-29

**Authors:** Lawrence X. Cai, Yohei Tanada, Gregory D. Bello, James C. Fleming, Fariz F. Alkassis, Thomas Ladd, Todd Golde, Jin Koh, Sixue Chen, Hideko Kasahara

**Affiliations:** 1 Department of Physiology and Functional Genomics, University of Florida, College of Medicine, Gainesville, FL 32610 USA; 2 Department of Neuroscience, University of Florida, College of Medicine, Gainesville, FL 32610 USA; 3 Proteomics and Mass Spectrometry, Interdisciplinary Center for Biotechnology Research (ICBR), University of Florida, Gainesville, FL 32610 USA; 4 Department of Biology, Genetics Institute, Plant Molecular and Cellular Biology Program, University of Florida, Gainesville, FL 32610 USA

**Keywords:** Molecular biology, Cardiovascular biology

## Abstract

Cardiac contractility is enhanced by phosphorylation of myosin light chain 2 (MLC2) by cardiac-specific MLC kinase (cMLCK), located at the neck region of myosin heavy chain. In normal mouse and human hearts, the level of phosphorylation is maintained relatively constant, at around 30–40% of total MLC2, likely by well-balanced phosphorylation and phosphatase-dependent dephosphorylation. Overexpression of cMLCK promotes sarcomere organization, while the loss of cMLCK leads to cardiac atrophy *in vitro* and *in vivo*. In this study, we showed that cMLCK is predominantly expressed at the Z-disc with additional diffuse cytosolic expression in normal adult mouse and human hearts. cMLCK interacts with the Z-disc protein, α-actinin2, with a high-affinity kinetic value of 13.4 ± 0.1 nM through the N-terminus region of cMLCK unique to cardiac-isoform. cMLCK mutant deficient for interacting with α-actinin2 did not promote sarcomeric organization and reduced cardiomyocyte cell size. In contrast, a cMLCK kinase-deficient mutant showed effects similar to wild-type cMLCK on sarcomeric organization and cardiomyocyte cell size. Our results suggest that cMLCK plays a role in sarcomere organization, likely distinct from its role in phosphorylating MLC2, both of which will contribute to the enhancement of cardiac contractility.

## Introduction

Actin-myosin cross-bridge formation is fundamental for cardiac contraction. During this process, phosphorylation of myosin light chain 2 (MLC2) located at the neck region of myosin heavy chain potentiates the force and rate of cross-bridge recruitment^1,2^. The level of MLC2 phosphorylation is maintained relatively constant, around 30–40% of total MLC2, by well-balanced phosphorylation and phosphatase-dependent dephosphorylation in normal hearts both in humans and mice^3–7^. Elimination of cMLCK in mice using homozygous germline and inducible mouse models leads to heart failure^7–10^. In particular, adult-inducible knockout mouse models demonstrate rapid and progressive heart failure with cardiac atrophy^10,11^.

cMLCK proteins are composed of roughly 3 different domains; the amino terminal domain unique to the cardiac isoform without homologies to other MLCKs, such as smooth muscle or skeletal types, followed by the conserved catalytic and regulatory domains^6,12^. In mouse neonatal cardiomyocytes, cMLCK is diffusely localized in the cytoplasm; however, in some areas, striated staining is observed. In these areas, unexpectedly, cMLCK does not colocalize with its substrate MLC2^6^. This finding suggests that phosphorylation of MLC2 would be a part of the function of cMLCK, or this localization would be critical to maintain phosphorylation of MLC2 around 30–40%.

Kinase-independent functions in smooth-muscle MLCK have been well established in cytoskeletal organization, cell migration, aggregation, and cell membrane tension, primarily due to the F-actin binding through a repeat motif (DFRXXL) located in the amino terminal domain unique to smooth-muscle MLCK^13–15^. cMLCK, however, does not contain the putative F-actin-binding DERXXL motif^6^.

One of the major actin cross-linking proteins found in virtually all cell types is actinin. Actinins are dimeric proteins, composed of the actin binding domain, spectrin-like repeats, and two pairs of EF-hand motifs^16–20^. Mammals have four actinin genes, *ACTN1-4*. The *ACTN*2 gene encodes α-actinin2, which is the major Z-disc protein in skeletal and cardiac muscle. In addition to binding to actin, it also serves as a scaffold of many other Z-disc components, and may be involved in mechanical strain sensing and signaling through interacting proteins^18,21–24^; yet how α-actinin2 enhances F-actin bundling is not fully understood^18,20^.

In this study, we showed that cMLCK is predominantly expressed at the Z-disc with additional diffuse cytosolic expression in mouse and human adult hearts. We investigated whether cMLCK can associate with other proteins, and found that cMLCK directly interacts with a Z-disc protein, α-actinin2 with high affinity (K_D_ value in nM range).

## Results

### cMLCK is predominantly expressed at the Z-disc, not overlapping with its substrate MLC2v in mouse and human hearts

**Figure 1:**
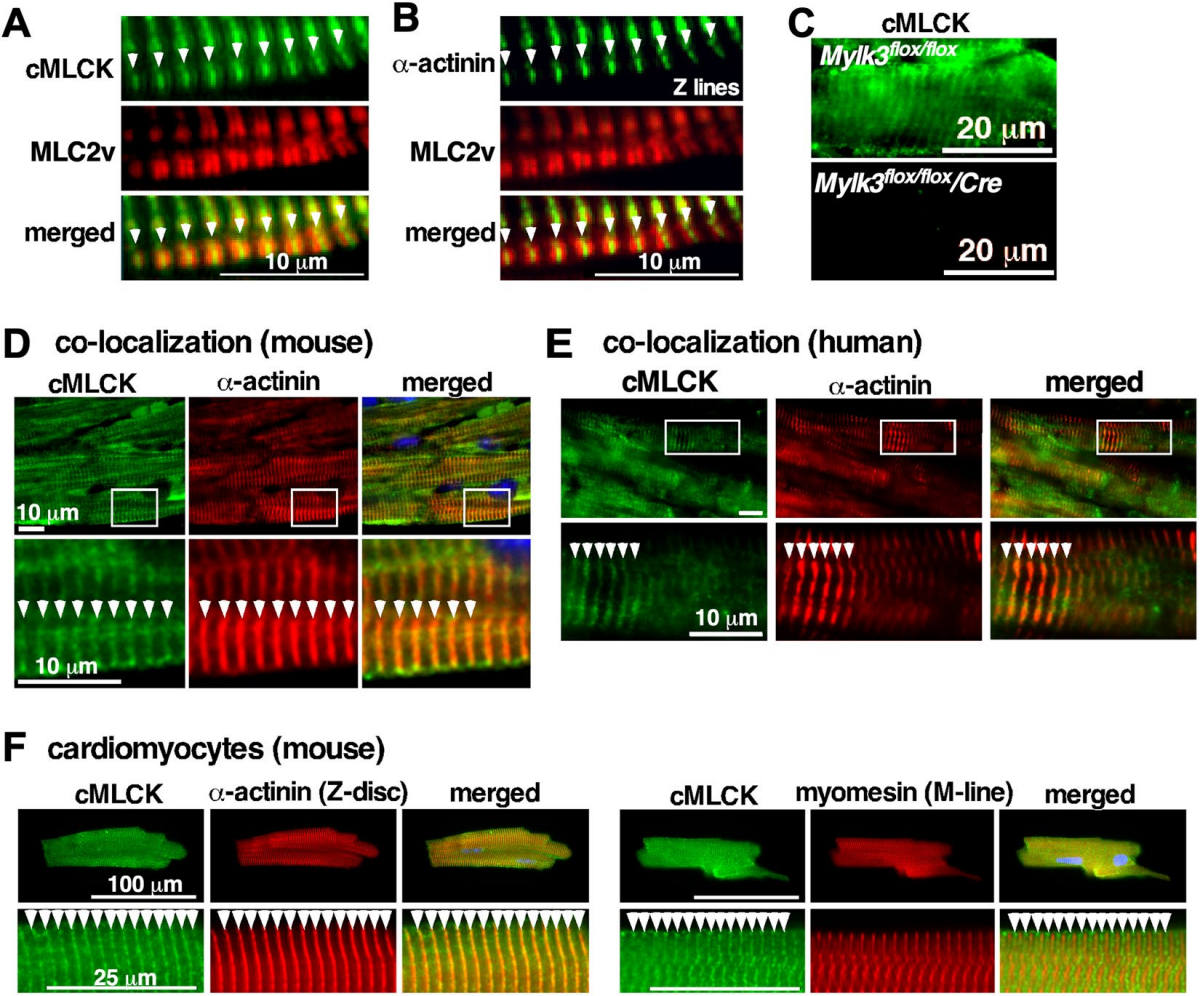
Expression of cMLCK proteins in mouse and human hearts. Representative co-immunostaining images of cMLCK, MLC2v and α-actinin2 using hearts from wild-type (**A**,**B**) or conditional *Mylk3* knockout mice (**C**). Co-immunostaining of cMLCK and α-actinin2 in wild-type mouse (**D**) and human hearts without apparent disease (**E**). (**F**) Co-immunostaining of cMLCK and α-actinin2 (left panel) and cMLCK and M-line protein myomesin (right panel) in isolated mouse adult cardiomyocytes. Bars = 10 µm in A, B, D, E; 20 µm in C; 100 or 20 µm in F.

cMLCK is the predominant kinase to phosphorylate MLC2 *in vivo* demonstrated in loss of function cMLCK mutant mice^7–10^. In neonatal cardiomyocytes, we previously showed that cMLCK is diffusely expressed in the cytoplasm, but it occasionally forms striation without overlapping with MLC2^6^. Striated cMLCK staining was predominant in adult mouse hearts with the high-intensity areas not overlapping with MLC2 (Fig. 1A, cMLCK in green, MLC2v in red), but overlapping with α-actinin2 (Fig. 1B). The specificity of cMLCK staining was shown in Fig. 1C. Further, co-immunostaining of cMLCK and α-actinin2 confirmed overlapping localization in mouse and human hearts, as well as in isolated adult mouse cardiomyocytes (Fig. 1D–F). In contrast, co-immunostaining of cMLCK and M-line protein myomesin did not show overlapping localization in isolated adult mouse cardiomyocytes (Fig. 1F, myomesin). Overall, in adult mouse and human hearts, cMLCK is predominantly expressed at the Z-disc with additional diffuse expression in the sarcomere.

### Interaction between cMLCK and Z-disc protein α-actinin2

**Figure 2:**
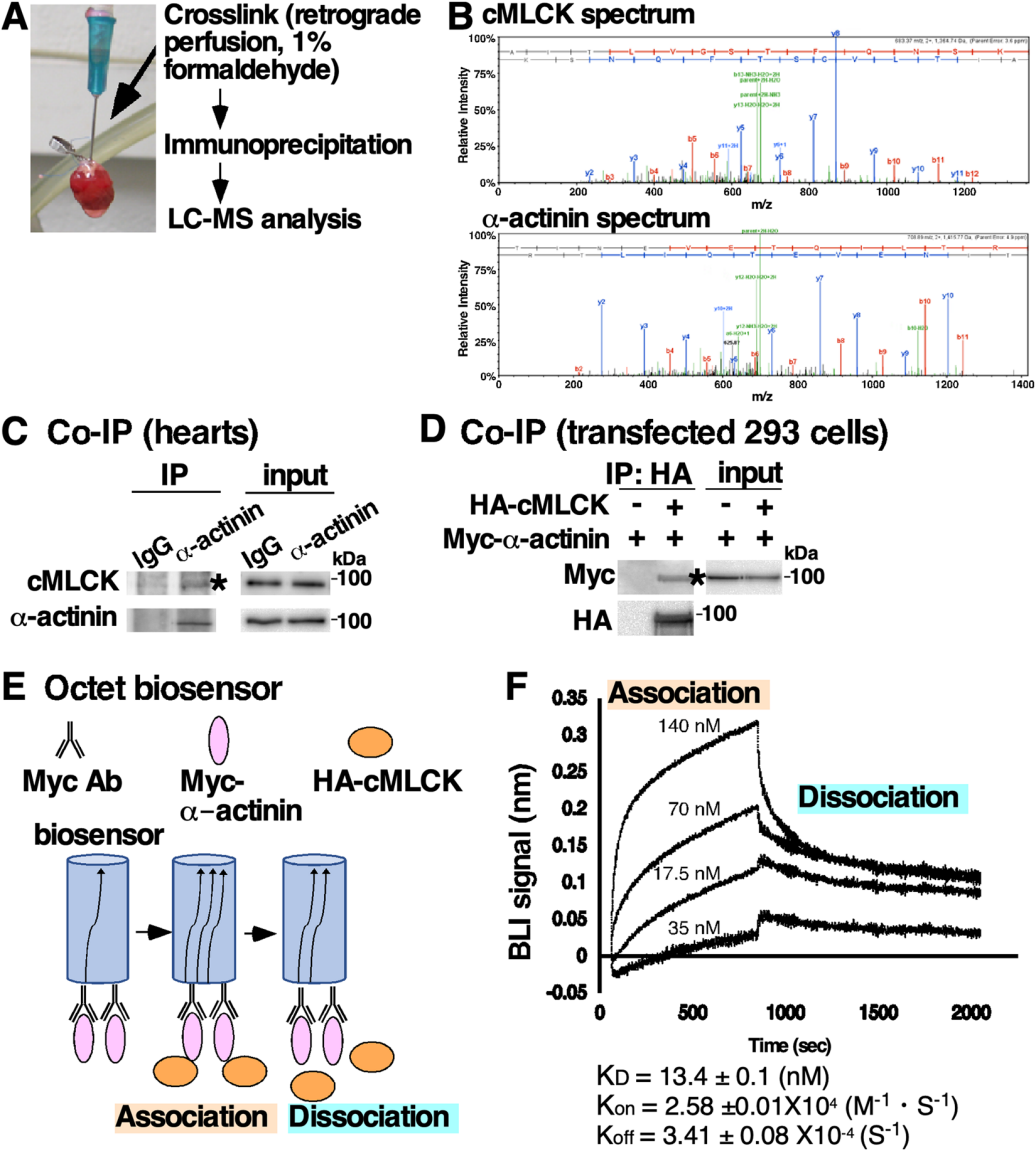
Interaction between cMLCK and α-actinin2. (**A**) A schematic of screening methodology to identify proteins interacting with cMLCK. (**B**) Representative mass spectra for cMLCK and α-actinin2 showing relative intensity (y-axis) and mass-to-charge ratio (m/z, x-axis). Co-immunoprecipitation of cMLCK and α-actinin2 using mouse heart lysates (**C**) and 293 cell lysates expressing exogenous HA-cMLCK and/or Myc-α-actinin2 (**D**). (**E**) A schematic of experiment for analysis of binding kinetics of cMLCK and α-actinin2 using biolayer interferometry (BLI). (**F**) Representative data showing association and dissociation kinetics of cMLCK to α-actinin2 using four different cMLCK concentrations. Mean values ± S.E.M (n = 2).

To understand potential functions of cMLCK localizing to the Z-disc, we screened proteins interacting with cMLCK *in situ*. Hearts isolated from transgenic mice expressing HA-tagged cMLCK^7^ were perfused with formaldehyde, followed by immunoprecipitation of heart lysates to obtain HA-tagged cMLCK and crosslinked proteins. Components of the protein complex were analyzed by liquid chromatography tandem mass spectrometry (LC-MS/MS) after digesting with trypsin (Fig. 2A). With 100% confidence and ~40% coverage, α-actinin2 was identified in the protein complex with cMLCK. Representative mass spectra of cMLCK and α-actinin2 were shown in Fig. 2B. In the protein complex, cardiac actin and CSRP3 (cysteine and glycine rich protein 3, MLP) were detected in LC-MS/MS analysis with 93–99% confidence and 15% and 6% coverage respectively (Supplemental Fig. 1). Because of high confidence and coverage, interaction between cMLCK and α-actinin2 was further analyzed, and confirmed by co-immunoprecipitation (co-IP) using mouse heart lysates, as well as HEK293 cell lysate expressing exogenous HA-cMLCK and Myc-α-actinin2 (Fig. 2C,D).

Quantitative binding affinity between cMLCK and α-actinin2 was analyzed using biolayer interferometry (BLI)(Fig. 2E). Anti-Myc-antibody was immobilized on a surface of the biosensor, to which purified Myc-α-actinin2 from HEK293 cells was bound. After washing excess of Myc-α-actinin2, four different concentrations of purified HA-cMLCK proteins were incubated to measure association kinetics, and then washed to measure dissociation kinetics. The kinetic values obtained by simultaneously fitting the association and dissociation responses from two independent experiments showed that the K_D_ value was 13.4 ± 0.1 nM (1.34 ± 0.1 × 10^−8^), K_on_ = 2.58 ± 0.01 × 10^4^ (1/Ms), and K_off_ = 3.41 ± 0.08 × 10^−4^ (1/s) (Fig. 2F).

### Domains required for interaction between cMLCK and α-actinin2

**Figure 3:**
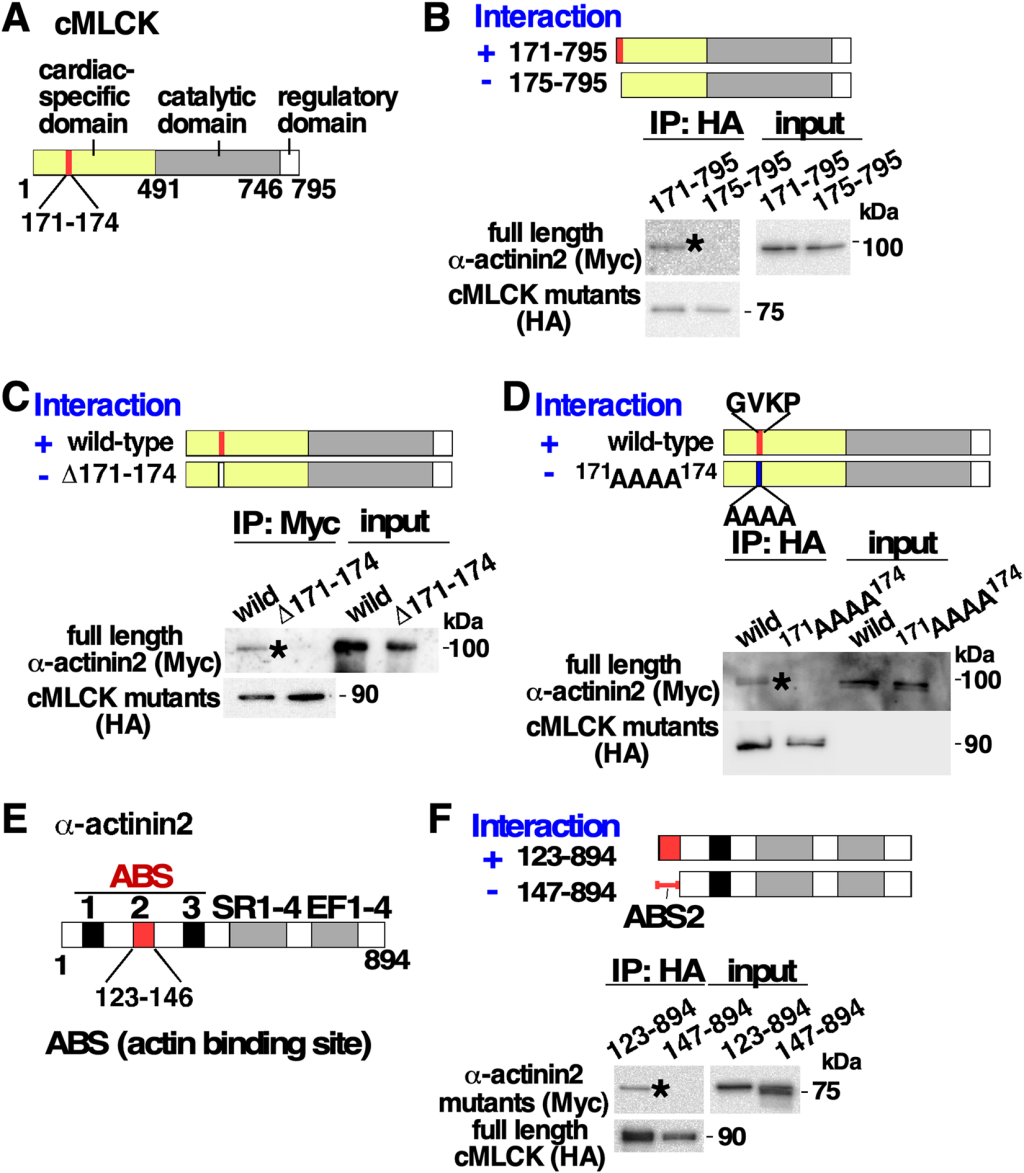
Identification of four amino acids located in the domain unique to the cardiac isoform required for interacting with α-actinin2. (**A**) Schematics of cMLCK protein including four amino acids 171–174 at the N-terminus region specific for cardiac isoform. (**B**) Two N-terminus deletion mutants, HA-tagged cMLCK(171–795) and cMLCK(175–795), were mixed with Myc-tagged full-length α-actinin2. Presence ( + ) and absence (−) of interaction between two proteins were indicated. (**C**) Internal deletion mutant of cMLCK (Δ171–174), and (**D**) four amino acid substituted mutants from ^171^DVKP^174^ to ^171^AAAA^174^ were mixed with Myc-tagged full-length α-actinin2. (**E**) Schematics of α-actinin2 protein including the second actin binding site. (**F**) Two Myc-tagged N-terminus deletion mutants of α-actinin2 with or without ABS2 domain were mixed with HA-full-length cMLCK. The additional results were available in Supplemental Fig. 1. ABS, F-actin binding site; SR, spectrin repeat; EF, EF hand motif.

cMLCK proteins are composed of roughly 3 domains; the amino terminal domain unique to the cardiac isoform, followed by the conserved catalytic and regulatory domains (Fig. 3A)^6,12^. To identify the regions required for interaction between cMLCK and α-actinin2, series of deletion mutants of HA-cMLCK were generated and mixed with full-length Myc-α-actinin2 *in vitro*. We found that the amino acid residues positioned between 171 and 174 (^171^GVKP^174^) within the cardiac-specific domain were required for binding to α-actinin2 (Fig. 3B, Supplemental Fig. 2A). The result was confirmed using internal-deletion (∆171–174) and missense cMLCK mutations (^171^AAAA^174^) (Fig. 3B–D).

α-actinin2 is composed of three actin binding sites (ABS) followed by spectrin-like repeats (SR) and two pairs of EF hand motifs (Fig. 3E). Serial deletion mutants showed that ABS2, positioned between amino acids 123 and 146, was required for binding to cMLCK (Fig. 3F, Supplemental Fig. 2B).

### Generation of kinase-deficient cMLCK mutant

**Figure 4:**
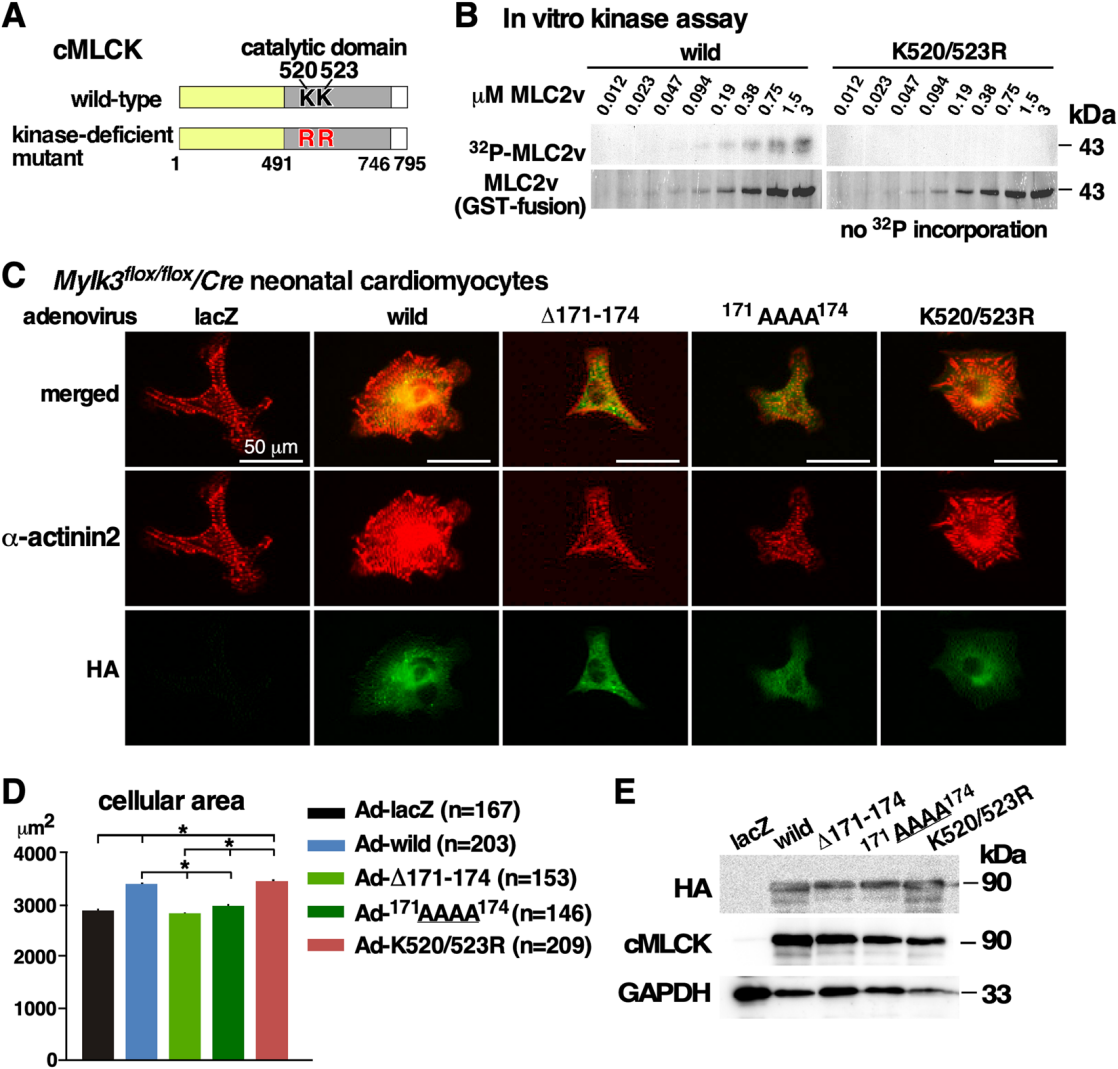
Generation of a kinase-deficient cMLCK mutant and effects of cMLCK mutations in neonatal cardiomyocytes. (**A**) Schematics of cMLCK protein including two amino acids ^520^Lys and ^523^Lys in the catalytic domain. (**B**) *In vitro* kinase assay using wild-type and kinase-deficient mutant cMLCK(K520/523R) using GST-fused MLC2v as substrates with different concentrations (0.012–3 µM). (**C**) Representative neonatal cardiomyocytes isolated from *Mylk3*^*flox/flox*^*/Cre* mice infected with five adenoviral constructs co-immunostained with α-actinin2 (red) and HA (green). (**D**) Summary of cellular area (µm^2^). Number of cardiomyocytes measured indicated on graph. (**E**) Representative Western blotting with HA, cMLCK and GAPDH antibodies using cardiomyocyte lysates. Mean ± S.E.M. Results were compared using one-way ANOVA followed by post-hoc test (SPSS ver. 25). *P < 0.05.

The catalytic domain of cMLCK has a high homology to skeletal and smooth muscle MLCKs^6^, which forms the conserved kinase domains consisting of ~250–300 amino acids residues forming 12 subdomains^25^. Based on the database^25^, an invariant lysine residue (likely K520 or K523 in mouse cMLCK) in the subdomain 2 is likely the key residue to anchor and orient ATP, interacting with glutamine (E536 in mouse cMLCK) localized to the subdomain 3. By substituting two lysine residues positioned at 520 and 523 in the ATP-binding binding site to arginine (K520/523R), a kinase-deficient mutant was generated (Fig. 4A,B).

### Effects of cMLCK mutants deficient for interacting with α-actinin2 and kinase-deficient mutant in neonatal cardiomyocytes

We previously showed that wild-type cMLCK enhances sarcomere organization in neonatal cardiomyocytes^6^. Neonatal cardiomyocytes with reduced endogenous cMLCK were isolated from *Mylk3*^*flox/flox/Cre*^ neonates delivered from tamoxifen-injected pregnant *Mylk3*^*flox/flox*^ female bred with *Mylk3*^*flox/flox/Cre*^ male. Adenovirus encoding ß-galactosidase (LacZ, control), wild-type and three cMLCK mutants, either deficient for interacting with α-actinin2 (∆171–174 and/or ^171^AAAA^174^) or deficient for phosphorylating MLC2 (K520/523R) were tested for sarcomere organization and cell size (Fig. 4C). Compared to Ad-lacZ infected cardiomyocytes, Ad-wild-type cMLCK enhanced sarcomere organization and increased cellular area size as expected (Fig. 4C,D). On the other hand, adenovirus encoding two mutants deficient for interacting to α-actinin2, ∆171–174 and ^171^AAAA^174^, did not change sarcomere organization and cellular area size compared to Ad-lacZ infected cardiomyocytes. When an adenovirus expressing kinase-deficient mutant, K520/523R, was infected, sarcomere organization was enhanced and cellular area size was increased. Of note, the cellular area was measured using isolated cardiomyocytes not attached to others. Representative Western blotting using HA and cMLCK antibodies relative to GAPDH using cardiomyocyte lysates showed a comparable level of expression from adenovirus encoding wild-type and three mutants of cMLCK (Fig. 4E).

### Effects of cMLCK mutants in adult cardiomyocytes

**Figure 5:**
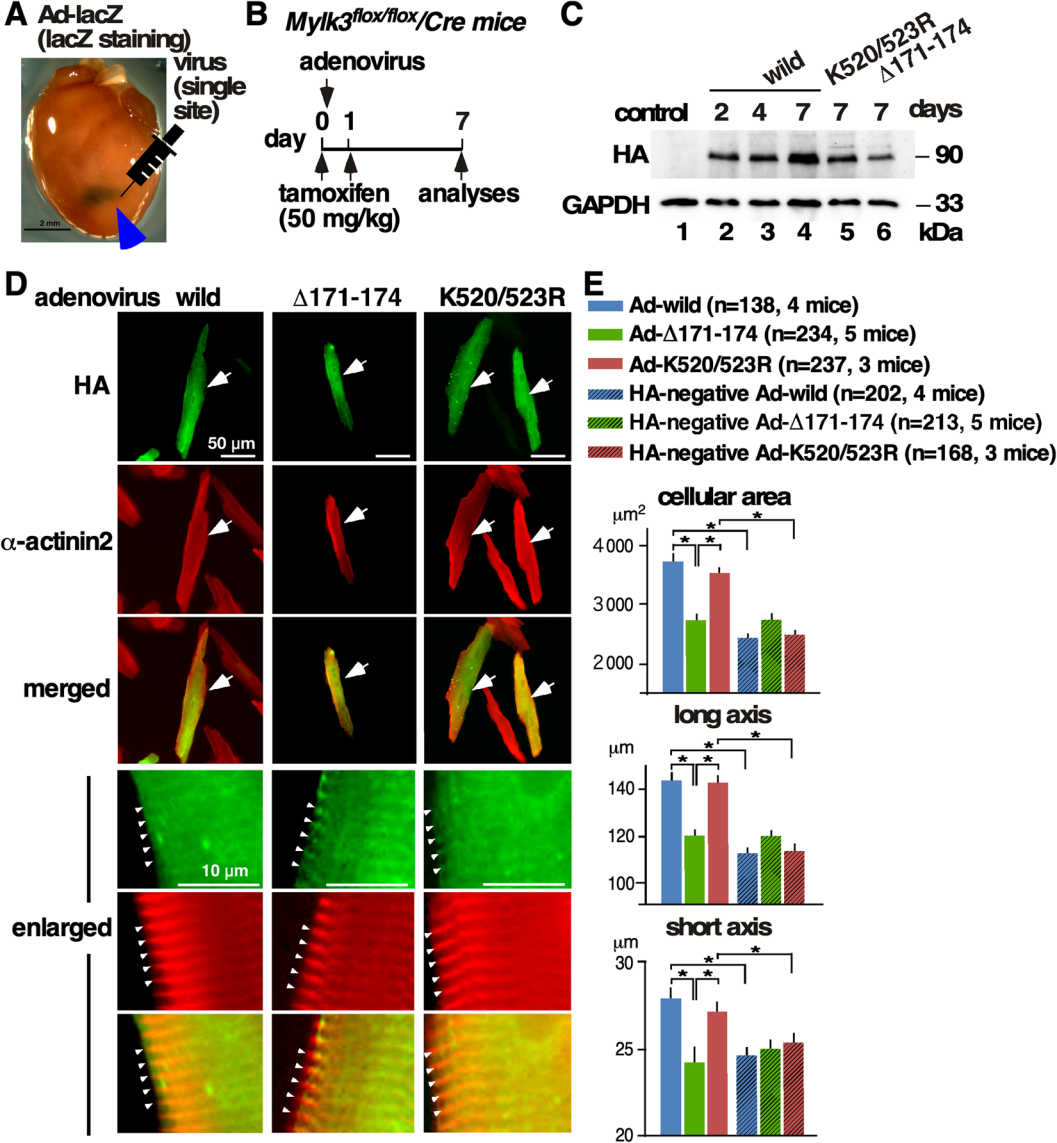
Effect of cMLCK mutations in adult cardiomyocytes. (**A**) Representative beta gal-stained heart 1 week after a single injection of adenovirus-lacZ (1 × 10^11^ viral particle in 30 µl) into the anterior LV wall. (**B**) Time schedule of experiments. (**C**) Representative Western blotting with HA and GAPDH antibodies using whole heart lysates at 2, 4 and 7 days after injection of adenovirus. (**D**) Representative adult cardiomyocytes isolated from *Mylk3*^*flox/flox*^*/Cre* mice infected with three viral constructs co-immunostained with HA (green) and α-actinin2 (red). Enlarged images were shown at the bottom. See Supplemental Fig. 3 for additional immunostaining images. (**E**) Summary of cellular area (µm^2^), long-axis (µm), and short-axis (µm). Number of cardiomyocytes measured was indicated on graph. Mean ± S.E.M. Results were compared using one-way ANOVA followed by post-hoc test (SPSS ver. 25). *P < 0.05.

It is difficult to maintain the rod-shaped morphology of adult cardiomyocytes in a cell culture system. To minimize the effects of endogenous cMLCK, purified adenovirus was directly injected at a single limited area of the left ventricular wall of inducible *Mylk3* adult knockout mice^10^. A representative lacZ-stained heart after a single injection of ad-LacZ virus was shown in Fig. 5A. The experimental timeline is shown in Fig. 5B; adenovirus was injected at a single site into the left ventricular wall (day 0), tamoxifen (50 mg/kg, i.p) was injected (days 0 and 1) and cardiomyocytes were isolated for measurement of cell size (day 7). Adenoviral-encoding wild-type cMLCK or mutants were detected on days 2 to 7 in the whole heart lysates (Fig. 5C).

Isolated cardiomyocytes were stained with HA antibody to detect those expressing either wild-type, Δ171–174, or K520/523R cMLCK. Cardiomyocytes expressing wild-type or kinase-deficient cMLCK mutant were significantly larger than those expressing cMLCK(Δ171–174) deficient for interacting with α-actinin2, as well as control HA-negative cardiomyocytes isolated from the corresponding hearts (HA-negative Ad-wild, or K520/523R, shown in corresponding hatched columns, Fig. 5D,E). There were no significant differences in cell size among HA-negative cardiomyocytes isolated from three groups, suggesting that the adenovirus injected into the limited LV wall did not affect majority of cellular size except for those expressing wild-type or kinase-deficient cMLCK mutant.

Enlarged epifluorescent images in Fig. 5D showed wild-type and cMLCK mutants colocalized with α-actinin2, indicating that cMLCK mutant either deficient for interacting with α-actinin2 or deficient for phosphorylating MLC2 did not change intracellular localization. Additional confocal microscopic images showing co-localization of cMLCK mutant deficient for interacting with α-actinin2 and α-actinin2 were shown in Supplemental Fig. 3. Overall, we showed that over-expression of wild-type and a kinase-deficient mutant showed similar effects on enhancement of sarcomeric organization and increase of cellular size, but not similar to a mutant deficient for interacting with α-actinin2.

## Discussion

MLC2 phosphorylation has been known to enhance myosin and actin interaction, leading to increased force power of cardiac contractility^1,2,4^. cMLCK is the predominant kinase to phosphorylate MLC2, however its expression in neonatal cardiomyocytes is diffuse in the cytoplasm with occasional striated patterns not overlapping with MLC2^6^. Striated cMLCK staining was more prominent in adult mouse and human hearts with the high-intensity areas not overlapping with MLC2 but overlapping with α-actinin2. Further, cMLCK directly interacts with a Z-disc protein α-actinin2 through the cardiac-specific domain. Kinetic analysis of binding between two proteins using biolayer interferometry showed the K_D_ value is around 10 nM range, which is considered to be moderate to high affinity^26^. cMLCK mutants deficient for interacting with α-actinin2 did not exhibit the ability to enhance sarcomere organization and increase cardiomyocyte cell size; while kinase-deficient mutant showed these effects similar to the wild-type cMLCK in neonatal and adult mouse cardiomyocytes.

Protein-protein interactions are critical for many cellular processes, including signaling and metabolism. In this study, we utilized perfusion of 1% formaldehyde for cross-linking proteins in hearts, in combination with LC-MS/MS for screening proteins interacting with cMLCK. This methodology has been promising to identify novel protein-protein and multiprotein interaction complexes^27–30^. The advantages of applying covalent cross-linking to intact tissues/organs include stabilizing *in vivo* protein-protein interactions and keeping the local spatio-temporal features which cannot be replicated *in vitro*. Formaldehyde cross-linking has several advantages. Due to its small size, formaldehyde can readily permeate cell walls and membranes, resulting in efficient cross-linking in a short-time period. It is considered a ‘zero-length’ cross-linker (although the actual arm distance is 2 Å), which is characteristic of only close-proximity associations, and minimizes non-specific protein interactions. Quenching with glycine and tris allows the sample to be directly used for co-immunoprecipitation. The reversibility of cross-linking by boiling in SDS-PAGE sample buffer is suitable for trypsin-digestion for MS analysis. Lower formaldehyde concentrations (such as 0.4–2% instead of 4% which is normally used for histology) and shorter reaction time (minutes instead of hours) will minimize non-specific cross-linking^27–30^.

α-actinin2 encoded by the *ACTN2* gene is the major Z-disc component in cardiomyocytes, playing a pivotal role in sarcomere organization by promoting actin bundling^31–40^. Missense mutations in *ACTN2*, indeed, cause dilated or hypertrophic cardiomyopathy^31–34,41^. α-actinin2 is composed of the actin binding domain followed by a short neck region, spectrin-like repeats and two pairs of EF hand motifs, and dimerizes^16–20^. The actin binding domain is composed of two calponin homology domains. When they are dissociated (opened), α-actinin2 has a higher affinity for F-actin compared to the closed form^20^. However, α-actinin2 does not exhibit a high affinity for actin *in vitro*^21^, suggesting that additional proteins will regulate this process *in vivo*. Indeed, α-actinin2 interacts with multiple proteins, such as CSRP3/MLP, phosphatidylinositol 4,5-bisphospate (PIP2), titin, α-actinin2-associated LIM protein (ALP), ZASP/Cypher, myopalladin and myotilitin^18,21–24,42^; yet how α-actinin2 enhances F-actin bundling is not fully understood^18,20^. Of note, we detected cardiac actin and CSRP3/MLP in the protein complex with cMLCK using LC-MS/MS analysis. However, differing from smooth muscle MLCK^13–15^, cMLCK did not directly bind to the cardiac actin using pull-down assays (data not shown).

To our knowledge, except for actin, cMLCK is the only protein which binds at ABS2. Our study showed that a cMLCK mutant deficient for interacting with α-actinin2 lost an ability to enhance sarcomere organization, however the underlying mechanism remains to be explored. cMLCK mutant deficient for interacting to α-actinin2 did not change its intracellular localization, indicating that localization at Z-disc and interaction with α-actinin2 occur independently.

Increased expression of cardiac MLCK induced sarcomere organization in neonatal cardiomyocytes^6^ as has been observed by overexpression of skeletal MLCK^43^. An ongoing question is why skeletal MLCK also enhances sarcomere organization despite the amino-terminus lacking homologies to cardiac MLCK and α-actinin2 binding sites. Ser19 phosphorylation of MLC2 leading to potentiation of the force and speed of contraction has been well studied *in vitro* and *in vivo*^1,2,4^, yet the phosphorylation level of ventricular MLC2 (MLC2v) is maintained relatively constant and low, around 30–40% of total MLC2. The majority of cMLCK localizes to the Z-disc and interacts with α-actinin2, which may regulate its catalytic activity by scaffolding cMLCK at the Z-disc.

In summary, we report for the first time cMLCK’s expression predominantly at Z-disc, where it interacts with α-actinin2. Wild-type cMLCK enhances sarcomere organization and increases cardiomyocyte cell size^6^. A cMLCK mutant deficient for phosphorylating MLC2 exhibits similar effects, but not mutants deficient for interacting with α-actinin2. These results suggest that cMLCK plays a role in sarcomere organization, likely separated from its role in phosphorylating MLC2, both of which will contribute to enhancement of cardiac contractility.

## Materials and Methods

### Mouse model and human heart samples

Generation of *Mylk3*^*flox/flox*^ with the *αMHCmerCremer* transgene^44^ backcrossing with C57BL/6 wild-type mice over 6 generations was reported previously^10^. To delete the floxed- *Mylk3* gene, tamoxifen (50–100 mg/kg body weight, ip) was injected into pregnant mice within 24 hr before delivery to generate inducible neonatal *Mylk3* knockout mice, or 50 mg/kg/day for 2 consecutive days to generate adult inducible *Mylk3* knockout mice^10^. Mice were euthanized either by inhalation of overdose isoflurane (approximately 5%), or CO_2_, followed by physical methodologies (cervical dislocation or decapitation). Mouse adult cardiomyocytes were isolated by perfusion of collagenase, followed by percoll gradients as described^7^. All animal experiments were performed with approval from the University of Florida Institutional Animal Care and Use Committee, which conforms to the NIH guidelines. Human heart samples without apparent disease were obtained from the National Disease Research Interchange, which prides itself on a strict adherence to government regulations and guidelines regarding informed consent and donor confidentiality. The informed consent was obtained from any donor of human tissue (or the next-of-kin thereof) for the use of that tissue for research. The use of tissues was approved by the institutional review board at University of Florida. All tissues were used in accordance with the Declaration of Helsinki.

### Western blotting, immunostaining, cell size measurement and histological analyses

Western blot analyses, immunostaining and immunoprecipitation were performed with the following antibodies: mouse cMLCK^6^, human cMLCK^7^, phospho-MLC2v (gift from Dr. N. Epstein, NIH)^45^, MLC2 (ALX-BC-1150-S-L005 Enzo Life Science), α-actinin2 (mouse monoclonal A7811 Sigma, rabbit polyclonal ab68167 Abcam), myomesin (mouse monoclonal, mMAC myomesin B4, Developmental Studies Hybridoma Bank), HA (mouse monoclonal 2367 Cell Signaling, rabbit monoclonal 3724 Cell Signaling); HA-agarose (26182 Pierce); HA-POD (12013819001 Sigma), Myc (mouse monoclonal 2276 Cell Signaling, rabbit monoclonal 2278 Cell Signaling); biotinylated Myc (B7554 Sigma); Myc-agarose (20168 Pierce); Myc-POD (11814150001 Sigma), and GAPDH (MAB374 MilliporeSigma).

Fluorescent staining for the heart sections was imaged using either Leica TCS-SP2 Laser Scanning Confocal Fluorescent Microscope System or an epifluorescent ZEISS Axiovert200M. Fluorescent staining of cardiomyocytes infected with different cMLCK mutants was performed side by side, followed by imaging with the same exposure time below the level of saturation using a ZEISS Axiovert200M. Cell size was measured using fluorescent images of neonatal cardiomyocytes or HA-positive adult cardiomyocytes using ImageJ. Mouse hearts were stained with beta-galactosidase substrate, X-gal, and were photographed as described^46^.

### Protein crosslinking followed by liquid chromatography tandem mass spectrometry (LC-MS/MS)

Hearts isolated from adult transgenic mice expressing HA-tagged cMLCK^7^ were hanged to the langendorff system and perfused with 1% formaldehyde for 10 min at room temperature. The heart lysates immunoprecipitated with HA-agarose were digested in-gel with trypsin, and analyzed via LC-MS/MS as described^47^ (Proteomics and Mass spectrometry core, ICBR, UF). Briefly, the excised gel bands were washed with equal volumes of 100 mM ammonium bicarbonate and acetonitrile until the gel pieces were destained. The samples were treated sequentially with 10 mM dithiothreitol for 30 min at 37 °C and 40 mM iodoacetamide in the dark for 1 h. The samples were digested in 10 ng/µL trypsin in 50 mM ammonium bicarbonate overnight. Tryptic peptides were desalted with ZipTip (MilliporeSigma).

The peptides were loaded onto an EASY-nLC 1200 liquid chromatography system. The flow rate was set at 250 nL/min with solvent A (0.1% formic acid in water) and solvent B (0.1% formic acid and 99.9% acetonitrile). Separation was conducted using the following gradient: 2–35% of B over 0–45 min, 35–98% of B over 45–46 min, and isocratic at 98% of B over 46–60 min. The full MS1 scan was performed on an Orbitrap Fusion Tribrid instrument equipped with a nano-electrospray source (Thermo Fisher Scientific) at 120,000 resolution from 350 to 2000 m/z. The AGC target was 2e^5^ and the maximum injection time was 50 ms. Peptides bearing 2–6 positive charges were selected for fragmentation with an intensity threshold of 1e^4^. Dynamic exclusion of 15 s was used to prevent resampling of high-abundance peptides. For MS/MS, the quadrupole isolation window was 1.3 Dalton. The cycle time was 1 second, and collision-induced dissociation was done at 35% of normalized collision energy. The AGC target was 1e^4^, and the maximum injection time was 35 ms.

The MS/MS data were analyzed using Mascot (Matrix Science, London, UK; version 2.4.1). Mascot was set up to search the IPI human database with the digestion enzyme trypsin, fragment ion mass tolerance of 1 Dalton and parent ion tolerance of 10.0 PPM. Carbamidomethyl of cysteine was specified as a fixed modification. Gln- > pyro-Glu of the n-terminus, deamidation of asparagine, glutamine and arginine, and oxidation of methionine were specified as variable modifications. Scaffold (version 4.2.1, Proteome Software Inc., Portland, OR) was used to validate MS/MS based peptide and protein identifications. Peptide identifications were accepted if they could be established at greater than 95.0% probability by the Peptide Prophet algorithm^48^. Protein identifications were accepted if they could be established at greater than 95.0% probability^49^ and contained at least one unique peptide.

### Co-immunoprecipitation

Hearts or HEK293 cells were lysed by sonication in lysis buffer (25 mM HEPES, 100 mM NaCl, 5 mM MgCl_2_, 1 mM DTT, 1% triton, 10% glycerol, Complete protease inhibitor cocktail, pH7.6, Sigma). Supernatants were precleaned using protein G agarose, precipitated with HA-agarose, and extensively washed with lysis buffer. Proteins were extracted from HA-agarose using SDS-PAGE buffer, and subjected to SDS-PAGE and Western blotting.

### Octet biosensor

Protein interaction rates (K_D_, K_on_, K_off_) were measured using bio-layer interferometry through the Octet RED384 instrument (ForteBio). All the experiments were performed at 30 °C, with constant shaking at 1000 rpm to measure kinetics for interaction and dissociation. Data acquisition was carried out using the ForteBio Octet Data Acquisition 9.0.0.49 software, and data analysis was conducted using the ForteBio Data Analysis 9.0 software.

Briefly, the protective coating was removed from the Dip and Read Streptavidin Biosensors (18-0009, ForteBio) using 200 μl of hydration solution (20 mM Tris, 500 mM NaCl, 0.002% Tween, pH 7.3) in a 96 clear well plate (Greiner Bio-One reference 655801). Plate temperature was set at 30 °C, and the plate was shaken for ten minutes prior to bio-layer interferometry kinetics measurement. The plate was set to shake. The experiment used the Standard kinetic acquisition rate setting.

Biosensors were dipped into solutions at different stages in a Black Tilted-bottom microplate (ForteBio reference 18,0019). Biosensors were first soaked with an assay buffer (20 mM Tris, 150 mM NaCl, 0.002% Tween, pH 7.3) to establish a baseline signal. Biotinylated-Myc monoclonal antibody (B7554, Sigma-Aldrich) at a concentration 3.125 μg/ml was immobilized on the biosensor followed by a brief washing using the assay buffer. Next, biosensors were soaked with Myc-α-actinin2 protein (with concentration 10 μg/ml, 100 nM), followed by a brief washing using the assay buffer and by an association step in HA-MLCK solution of variable concentration (using a two-fold serial dilution starting from 14 μg/ml to 1.75 μg/ml), as well as a concentration of 0 μg/ml in order to perform background subtraction in data analysis. Dissociation was performed by reintroducing the biosensors back into the assay buffer.

The data was then interpreted using ForteBio Data Analysis 9.0 software for kinetics analysis. The biosensor dipped into the control 0 μg/ml HA-MLCK solution was used as a reference well for background subtraction. The y-axis was aligned to baseline, and inter-step correction was aligned to dissociation. Following Savitzky-golay filtering, the data was analyzed using the full step times for association and dissociation. K_D_, K_on_, and K_off_ were obtained using the global (full) group by color settings, with Rmax unlinked by sensor, under the 2:1 heterogeneous ligand analysis.

### Generation of cMLCK and actinin2 expression plasmid and adenovirus

pAdlox-HA-cMLCK expression vector was generated previously^6^. RNA isolated from neonatal mouse hearts were subjected to reverse transcription using random priming followed by PCR using four sets of specific primers and cloned into pAdlox plasmid DNA to generate pAdlox-Myc-α-actinin2. Deletion mutants for HA-tagged cMLCK and Myc-tagged α-actinin2 were generated by PCR mutagenesis using specific primers that include HA tag, 5′-ATGTACCCATACGATGTTCCAGATTACGCT-3′(encoding Met-Tyr-Pro-Tyr-Asp-Val-Pro-Asp-Tyr-Ala) or a Myc tag 5′-ATGGAACAGAAACTGATCTCTGAAGAAGACCTG-3′, encoding Met-Glu-Gln-Lys-Leu-Ile-Ser-Glu-Glu-Asp-Leu) at the amino-terminus.

The following primers were utilized for generation of internal and/or substitution mutants.

cMLCK kinase-deficient mutants: F: 5′-AGGCCTTGCACTGGCAGCCAGGATCATCAGAGTGAAGAACGTAAAGGAC-3′, R: 5′-GTCCTTTACGTTCTTCACTCTGATGATCCTGGCTGCCAGTGCAAGGCCT-3′, which will mutate Lys520 (AAG) to Arg (AGG) and Lys523 (AAA) to Arg (AGA).

cMLCK∆171–174 mutant: F: 5′- AAGGAGCAAGCAGAAGTTGCTAACCATGTACTGACTACAGGAGG-3′, R: 5′- CCTCCTGTAGTCAGTACATGGTTAGCAACTTCTGCTTGCTCCTT-3′.

cMLCK ^171^AAAA^174^ mutant: F: 5′- AAGGAGCAAGCAGAAGTTGCTGCAGCTGCGGCCAACCATGTACTGACTACAGGAGG-3′, R: 5′-CCTCCTGTAGTCAGTACATGGTTGGCCGCAGCTGCAGCAACTTCTGCTTGCTCCTT-3′.

Adenovirus encoding wild-type cMLCK and control Adlox-LacZ were generated previously^6^. The same methodology was used for generating additional adevenovirus encoding cMLCK mutants and Myc-α-actinin2.

### Kinase assay

A kinase assay was performed following our protocol^6^. Briefly, *in vitro* phosphorylation reactions were performed using recombinant HA-tagged MLCK proteins expressed in 293 cells. HA tagged-cardiac MLCK proteins were purified by HA-affinity columns, eluted with HA-peptides and dialyzed with kinase buffer (25 mM HEPES, 200 mM NaCl, 10 mM MgCl_2,_ pH 7.6). Approximately 0.15 µg/ml (1.7 nM) of cardiac MLCK and 0.14 mg/ml (3 mM) of GST-MLC2v with 2-fold serial dilution were mixed in 25 µl (final concentration 25 mM HEPES, 10 mM MgCl_2_, 5 mM DTT, 20 mM NaCl, 0.2% triton, 2% glycerol, 0.5 mg/ml BSA, 0.5 mM [*γ*-^32^P]ATP at 267 cpm/pmol, pH7.6) and incubated at 30 °C for 15 min. After termination of the kinase reaction by the addition of SDS-sample buffer, the samples were separated by 15% SDS-PAGE gel, stained with Coomassie blue and autoradiographed.

### Adenovirus purification, infection, and injection into left ventricular wall

Neonatal mouse cardiomyocytes were isolated from 2-day-old *Mylk3*^*flox/flox*^*/αMHCmerCremer* by trypsin digestion followed by Percoll gradient purification according to a protocol described previously with modifications^50^. Infection of adenovirus Adlox-MLCK (1–2 moi) and control Adlox-LacZ (1–2 moi) was performed in suspension immediately after purification followed by plating on laminin-coated plates or glass coverslips in medium (DMEM F-12, 5% new born calf serum, 0.5% of Insulin-Transferrin-Selenium, Thermofisher). Medium was changed after 24 hrs and followed by additional 48 hr incubation using our standard methodology.

Adenovirus was purified using ViraBind adenovirus purification kit (VPK-100, Cell Biolabs, Inc), and dialyzed against PBS. 1 × 10^11^ viral particles in 30 µl PBS was injected into mouse LV anterior wall at a single site using 31G needle. Adult cardiomyocytes were isolated by perfusion of collagenase followed by percoll gradient purification using our standard methodology^7,10,51^.

### Statistical analyses

Data presented are expressed as mean values ± S.E.M. Results were compared using one-way ANOVA followed by Fisher’s or Bonferroni’s post-hoc test (SPSS ver. 25). *P* < 0.05 was considered significant.

## Supplementary information

Dataset 1
