# Supplementary material for: Cardiac MLC2 kinase is localized to the Z-disc and interacts with α-actinin2
Source: Sci Rep. 2019 Aug 29;9:12580. doi: 10.1038/s41598-019-48884-w (PMC6715661; doi:10.1038/s41598-019-48884-w)
Supplement: Supplementary file 1 — Dataset 1 [file 41598_2019_48884_MOESM1_ESM.pdf]

Supplemental materials

Cardiac MLC2 kinase is localized to the Z-disc and interacts with α-actinin2

Lawrence Cai, Yohei Tanada, Gregory Bello, James Fleming, Fariz Alkassis, Thomas Ladd, Todd Golde, Jin Koh, Sixue Chen, and Hideko Kasahara

Supplemental Figure 1

Identification of two additional Z-line localized proteins in LC-MS/MS analysis. **(A)** List of the coverage, peptide sequences, unique peptides, peptide-spectra match (PSM), peptide confidence, and peptide masses, mass error in part per million (ppm), Mascot score, Expectation (Pval) for Cardiac actin (actin, alpha cardiac muscle) and CSRP3 (Cysteine and glycine-rich protein 3, MLP). Representative mass spectra for Cardiac actin **(B)** and CSRP3 **(C)** showing relative intensity (y-axis) and mass-to-charge ratio (m/z, x-axis).

A

Gene Annotation	Coverage	Peptide Sequence	PSMs	Peptide Confidence	Observed Mass (MS1)	Theoretical Mass	Calculated Mass	Charge status	ppm	Mascot Score	Expectation (Pval)
**Actin, alpha cardiac muscle 1**	15%	K.AGFAGDDAPR.A	3	100%	488.7301	975.4456	975.441	2+	4.75	44	4.00E-05
		K.DSYVGDEAQSKR.G	2	98%	677.8178	1353.6211	1353.616	2+	3.7	31	0.0008
		K.SYELPDGQVITIGNER.F	3	100%	895.9535	1789.8925	1789.885	2+	4.42	68	1.60E-07
		R.VAPEEHPTLLTEAPLNPK.A	2	100%	978.5302	1955.0457	1955.036	2+	4.8	36	0.00025
**Cysteine and glycine-rich protein 3**	6%	R.AATTSNPSKFSAK.F	1	99%	655.3435	1308.6725	1308.667	2+	3.9	35	0.00035

B Cardiac actin

R.AATTSNPSKFSAK.F

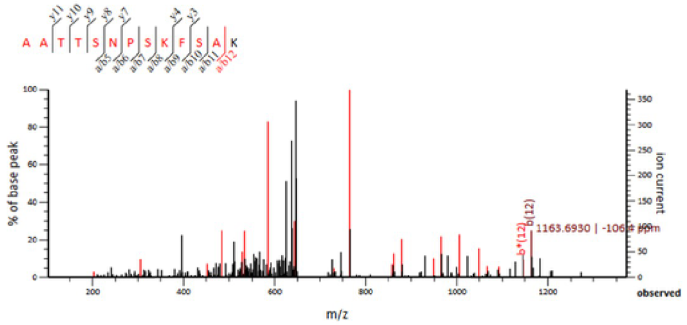

K.DSYVGDEAQSKR.G

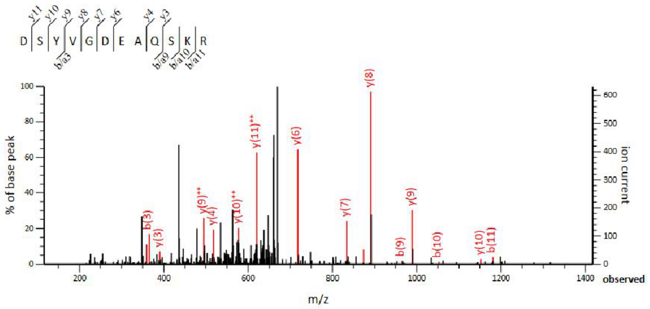

K.SYELPDGQVITIGNER.F

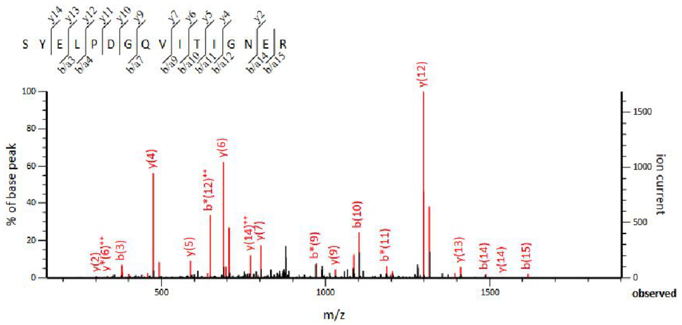

R.VAPEEHPTLLTEAPLNPK.A

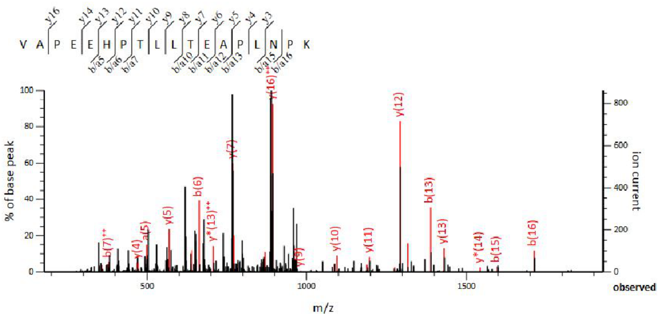

C CSRP3 (MLP)

R.AATTSNPSKFSAK.F

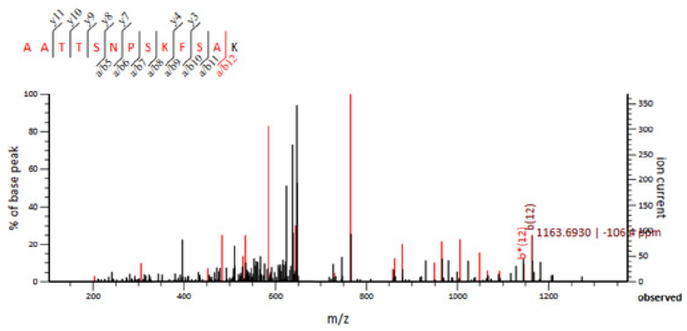

Supplemental Figure 2

Identification of domains required for interaction between cMLCK and α-actinin2. (**A**) Schematics of the cMLCK protein and the results of three independent experiments using series of cMLCK deletion mutants mixed with full-length α-actinin2. (**B**) Schematics of α-actinin2 protein and the results of experiments using series of α-actinin2 deletion mutants mixed with full-length cMLCK. Presence (+) and absence (-) of interaction between two proteins are indicated.

A cMLCK deletion mutants

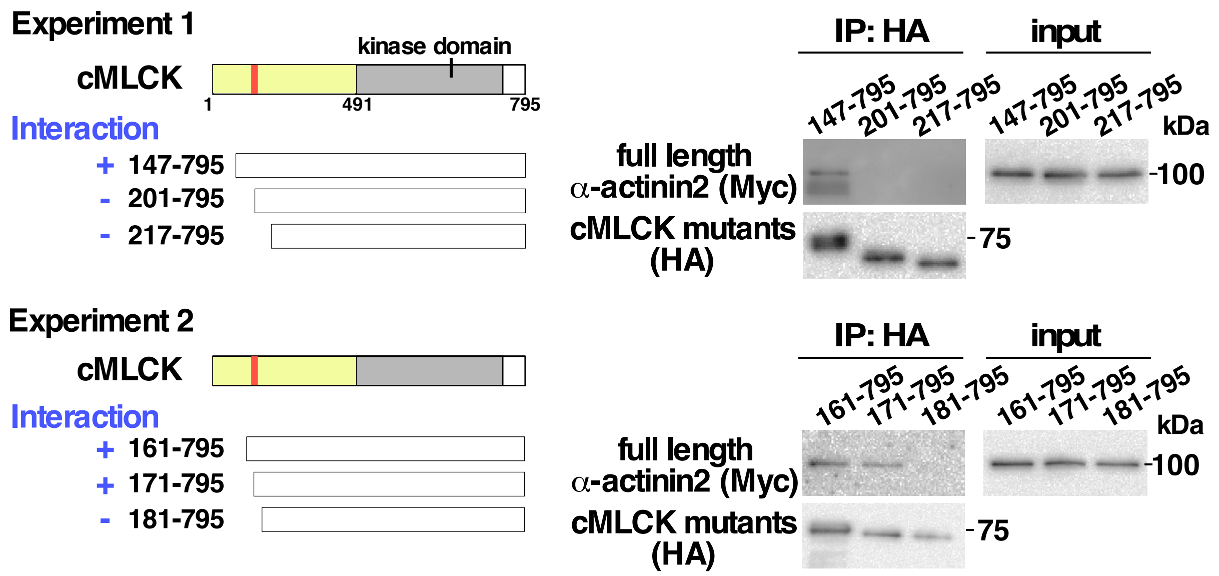

B α-actinin2 deletion mutants

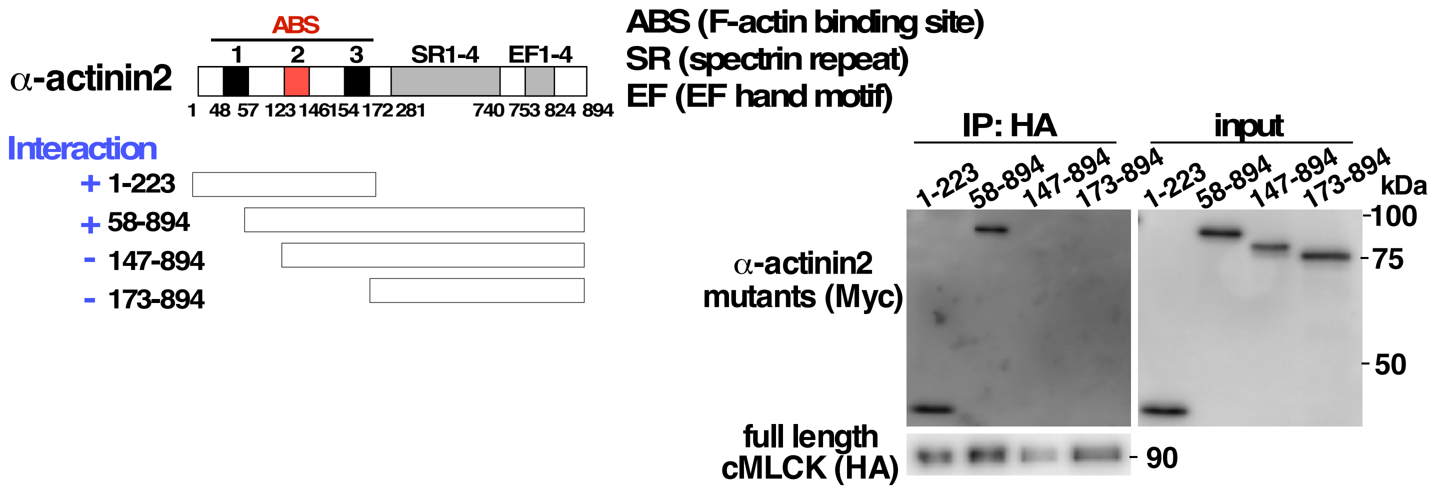

Supplemental Figure 3

Co-localization of HA-cMLCK(Δ171-174) and α-actinin2. Representative co-immunostained adult cardiomyocytes isolated from *Mylk3*$^{flox/flox}$*/Cre* mice infected with HA-cMLCK(Δ171-174) viral construct with HA (green) and α-actinin2 (red) and merged images obtained from a confocal microscope. Enlarged images were shown.

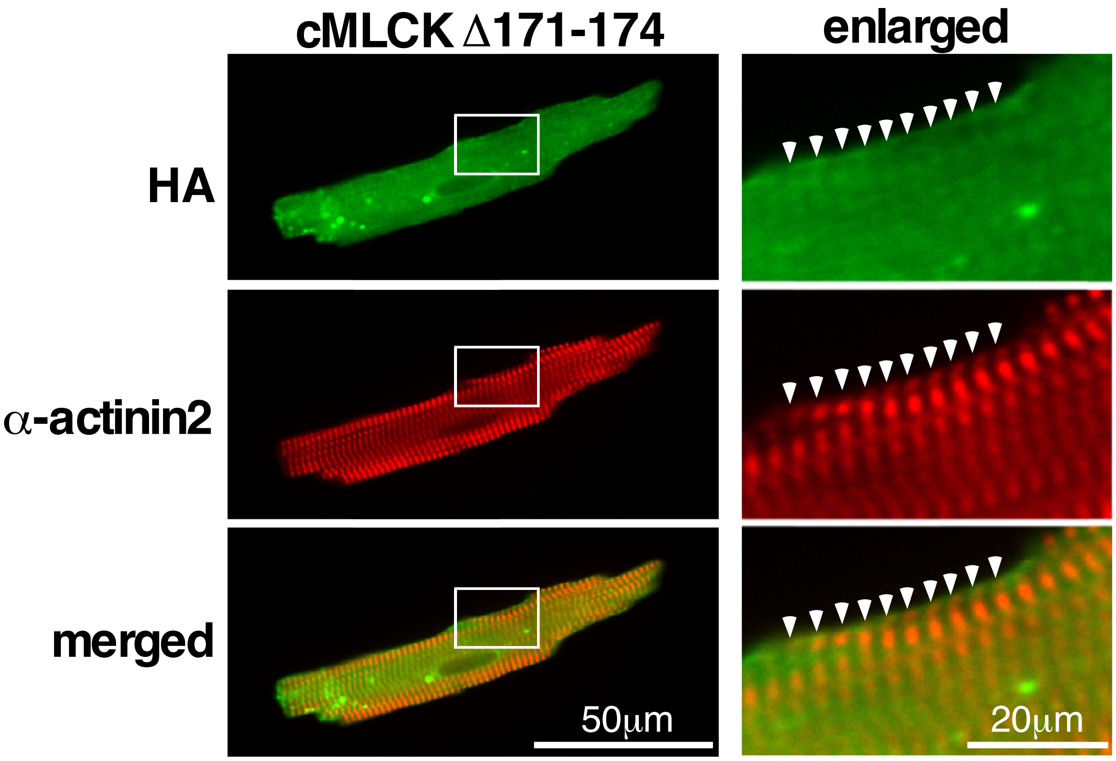

Supplemental Figure 4. Full-length blots used for Figure 2D.

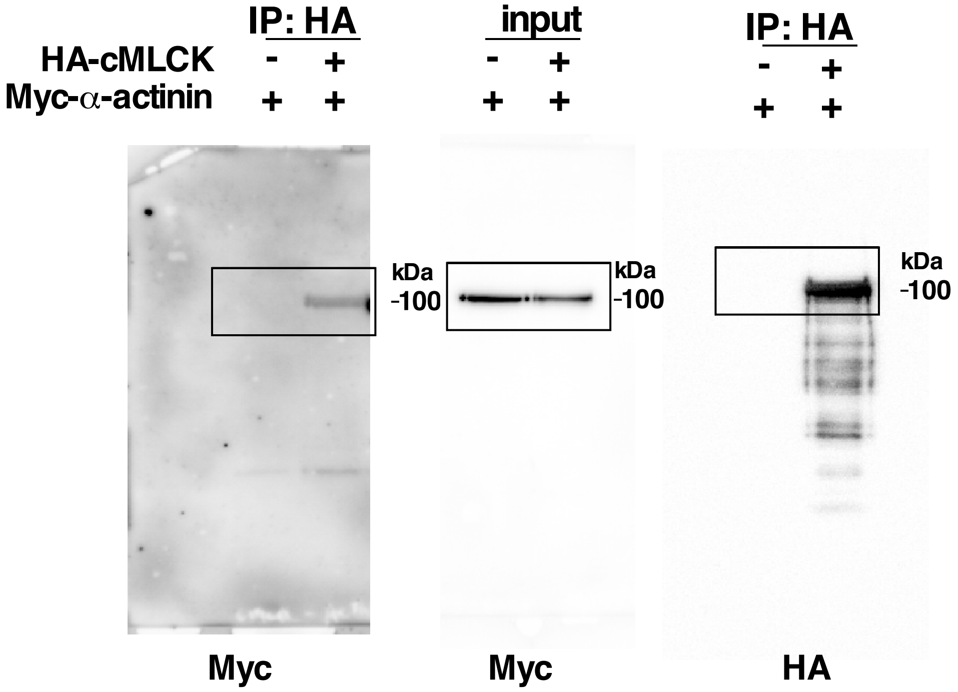

Supplemental Figure 5. Full-length blots used for Figure 3C.

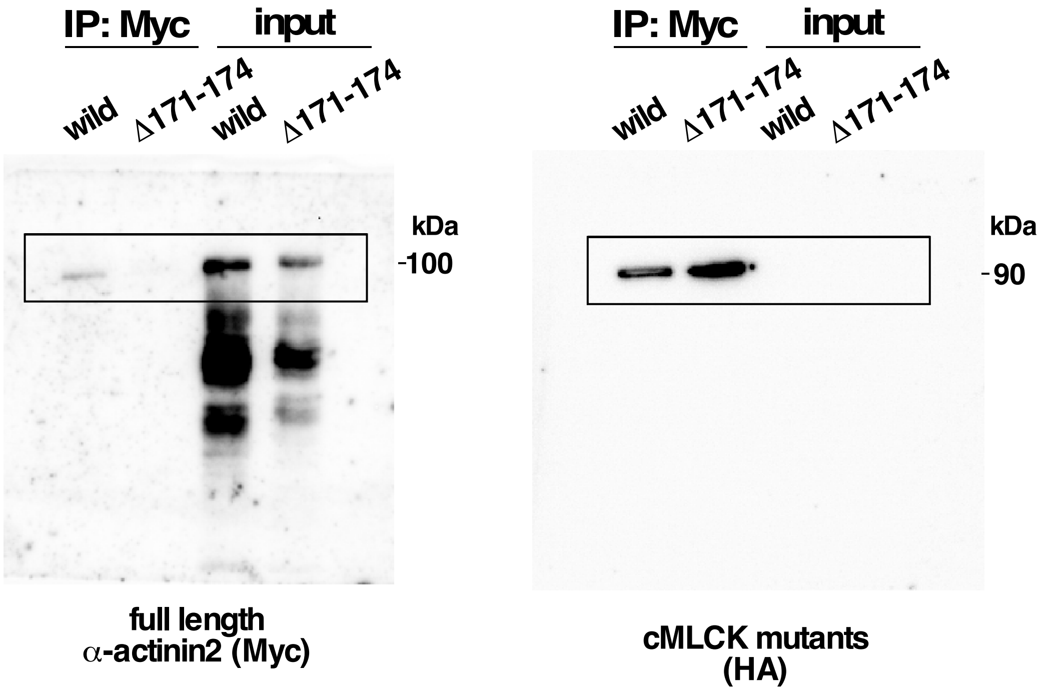

Supplemental Figure 6. Full-length blots used for Figure 3D.

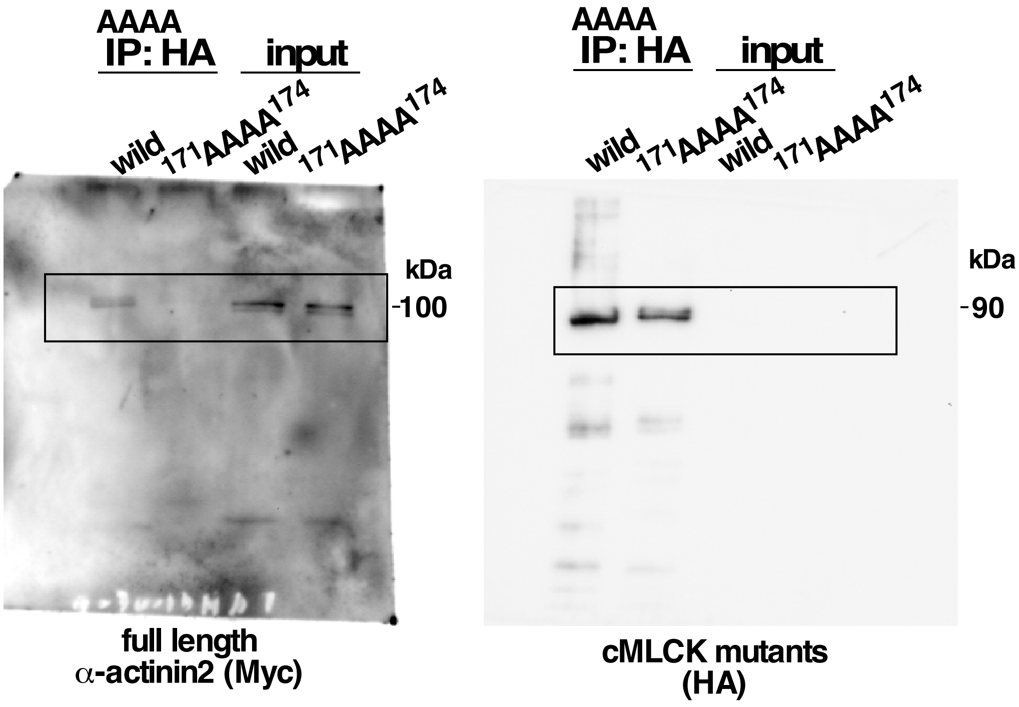

Supplemental Figure 7. Full-length blots used for Figure 3F.

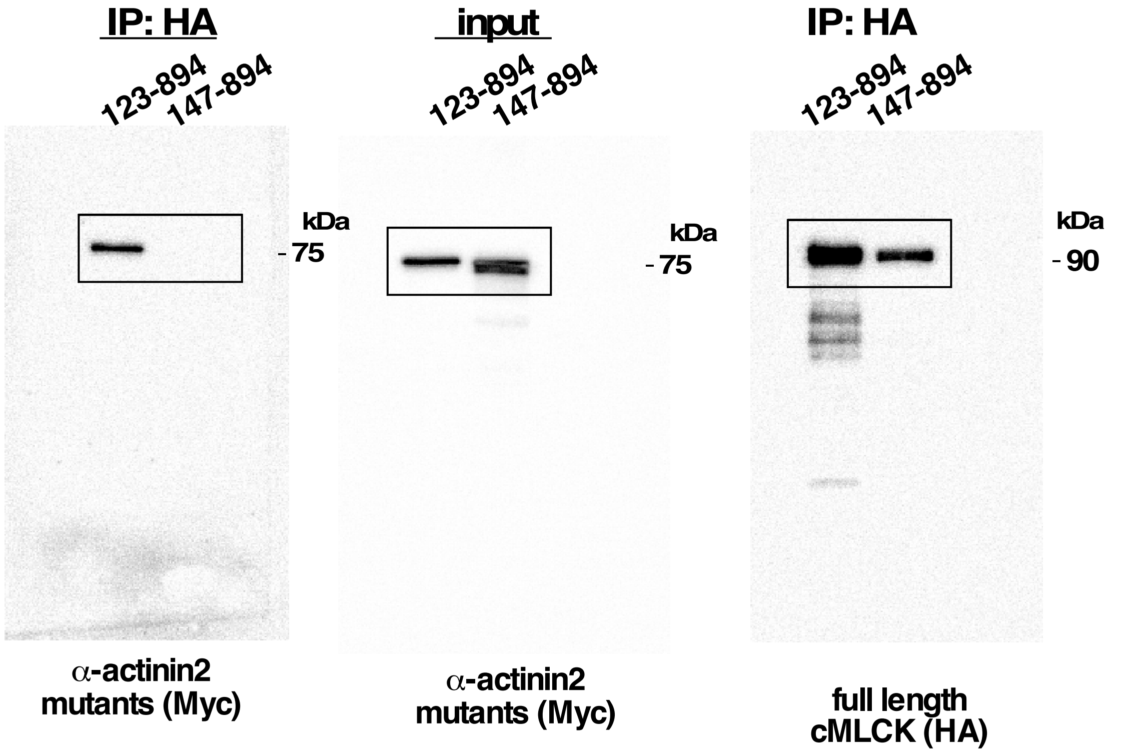

Supplemental Figure 8. Full-length autoradiographic and gel images used for Figure 4B.

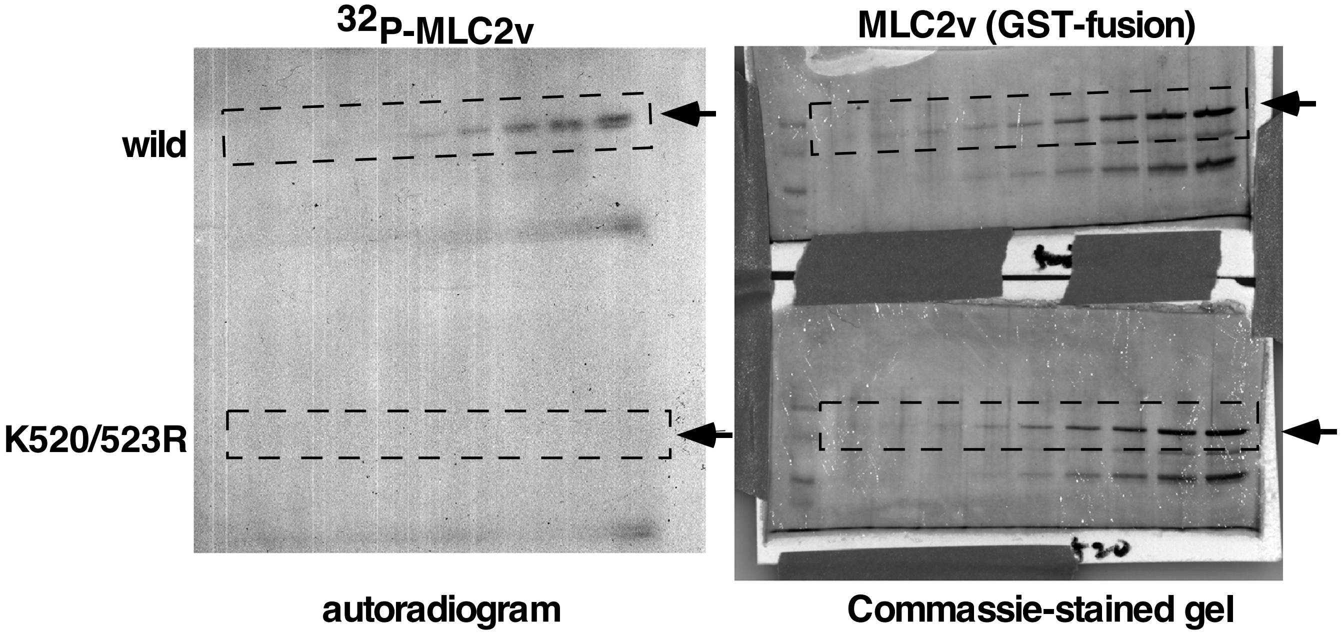

Supplemental Figure 9. Full-length blots used for Figure 4E.

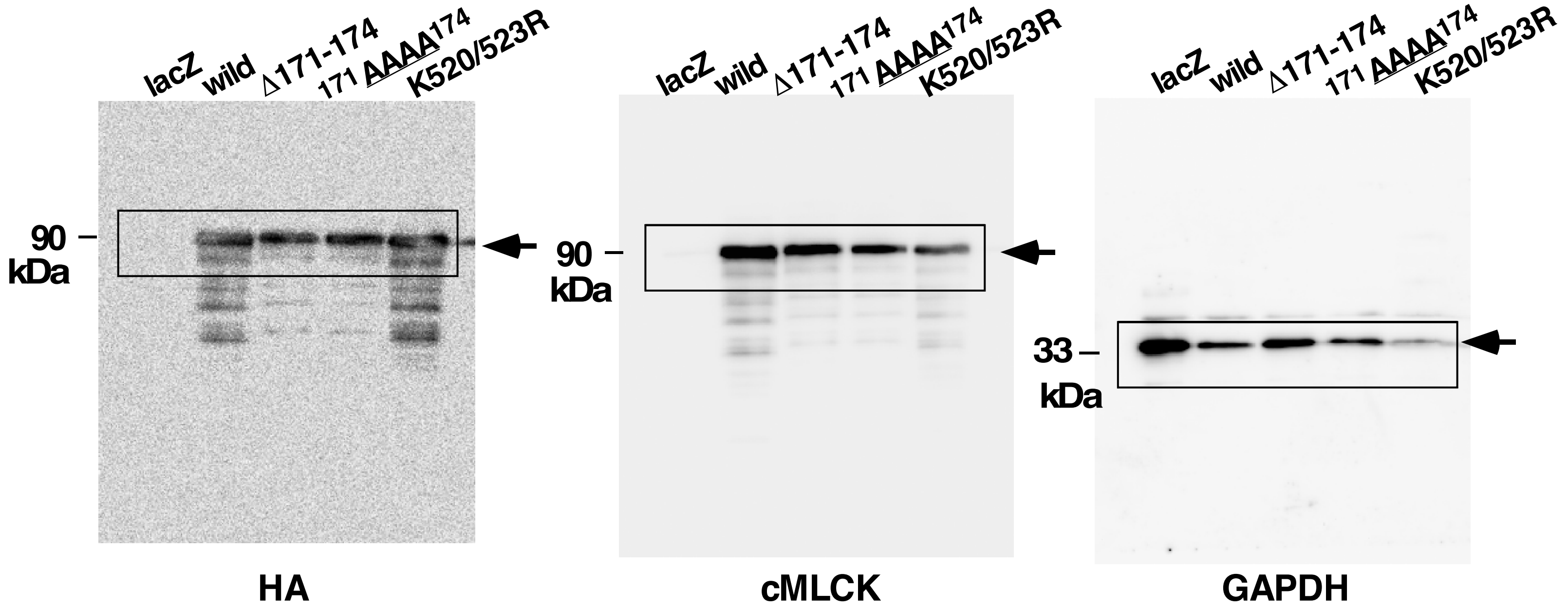

Supplemental Figure 10. Full-length blots used for Figure 5C.

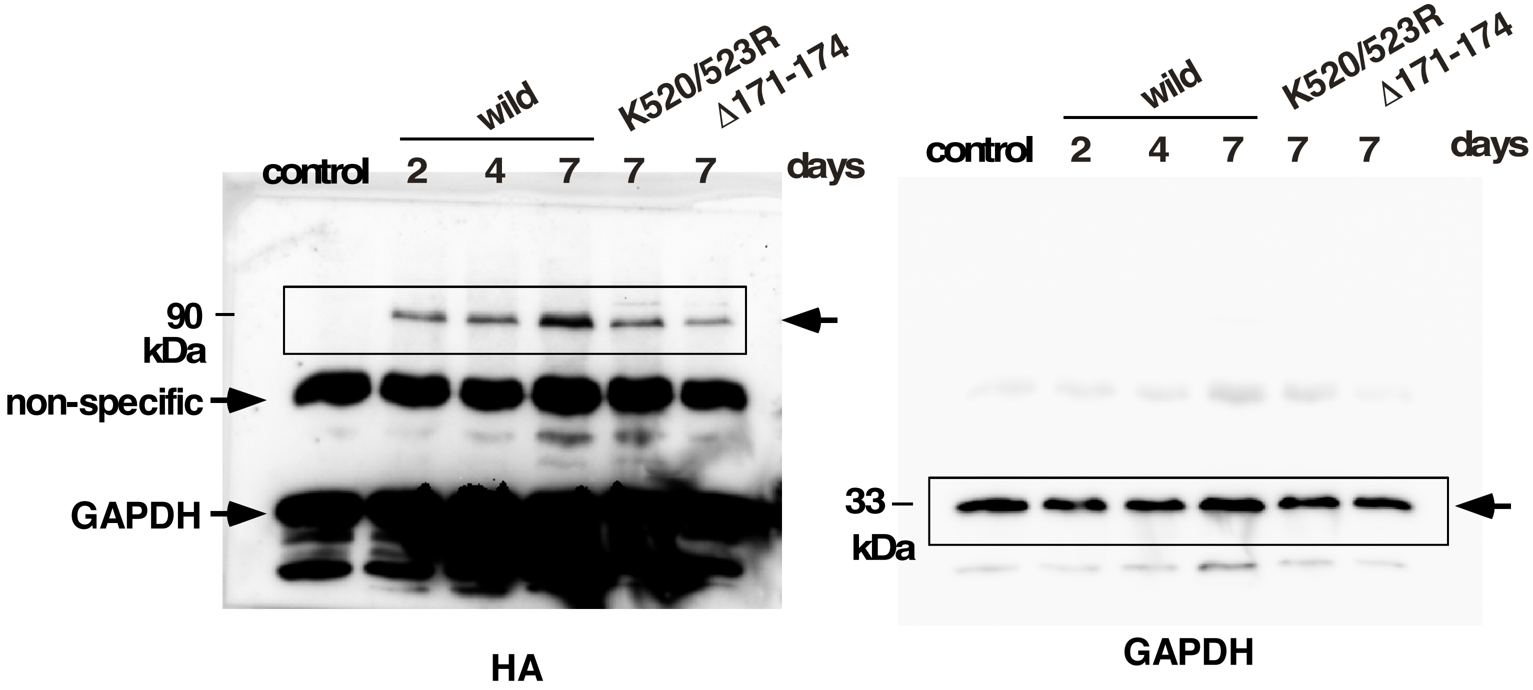

We were unable to find the full length blots used for Figures 2C and 3B, despite that we spent a large amount of time and efforts.
